# Physical Activity as a Mediator in the Relationship Between Body Image Perception and Low Mood in Adolescents

**DOI:** 10.3390/ijerph22020288

**Published:** 2025-02-15

**Authors:** Geiziane Leite Rodrigues Melo, Larissa Alves Maciel, Rafaela Espírito Santo, Caroline Brand, Cézane Priscila Reuter, Artūras Razbadauskas, Alona Rauckienė-Michaelsson, Cesar Agostinis-Sobrinho

**Affiliations:** 1 Health Research and Innovation Science Centre, Klaipeda University, 92294 Klaipeda, Lithuania; rafaela.santo@ku.lt (R.E.S.); alona.rauckiene-michaelsson@ku.lt (A.R.-M.); cesaragostinis@hotmail.com (C.A.-S.); 2 Postgraduate Program in Physical Education, Catholic University of Brasilia, Taguatinga 71966-700, Brazil; larimaciel@live.com; 3 IRyS Group, Physical Education School, Pontificia Universidad Católica de Valparaíso, Valparaíso 2340025, Chile; caroline.brand@pucv.cl; 4 Graduate Program in Health Promotion, University of Santa Cruz do Sul (UNISC), Santa Cruz do Sul 96815-900, Brazil; cezanereuter@unisc.br

**Keywords:** adolescence, body image, lifestyle, physical activity, mental health

## Abstract

Body image (BI) plays a critical role in mental health, with negative perceptions often linked to feelings of low mood. Physical activity (PA) has been shown to enhance self-acceptance and reduce negative emotions, suggesting it may help mitigate the impact of BI on low mood. This study examines the relationship between BI and low mood among adolescents, and explores the role of PA as a mediator in this relationship. The study had a cross-sectional design. Data were collected from 154,183 adolescents (average age 13.6 ± 1.6 years) across 43 countries, using the 2017/2018 Health Behaviour in School-aged Children (HBSC) survey. BI was assessed through self-perceived body size, low mood was measured on a scale from “about every day” to “rarely or never”, and PA was evaluated by the number of days per week participants engaged in at least 60 min of activity. The study used multinomial logistic regression and a mediation model to analyze the relationships of BI, PA, and BMI with low mood. The multinomial logistic regression showed that daily PA reduces the risk of low mood, especially with higher PA frequency, such as PA on 6 days (OR = 0.72) and PA on 5 days (OR = 0.86). Age, BMI, BI, and sex also influence low mood, with males showing lower odds (OR = 0.40 to 0.77), normal weight individuals having a reduced risk, and thinner individuals having lower odds of low mood (OR = 0.12 to 0.50), with PA partially mediating the relationship between BI and low mood (*p* < 0.001), contributing to 9% of the total effect. PA partially mediates the relationship between BI and low mood, with a direct negative impact of BI on low mood. In addition, girls, older adolescents, and those with negative BI and irregular PA have a higher risk of low mood.

## 1. Introduction

Mood disorders among adolescents represent a growing public health concern, often contributing to several mental health problems such as emotional distress, depression, anxiety, and others [1,2]. Children and adolescents are especially susceptible to mood disorders because adolescence is a time of fast emotional and psychological growth [3]. Body image (BI) perception has been shown to be a major determinant of mood during this period [4]. Additionally, BI has been identified as a global mental health concern, reflecting its pervasive impact across cultures and highlighting the necessity for public health interventions targeting this issue [5]. Low mood is more likely to emerge in adolescents with negative BI, such as those who believe they are overweight or “not fitting in” physically [6,7,8].

The current literature has shown that dissatisfaction with BI is strongly associated with low self-esteem, feelings of low mood, and clinical depression [9,10,11]. Furthermore, BI perception in children and adolescents significantly impacts their physical and mental health, influencing their development and overall well-being [12,13,14]. When children and adolescents perceive their bodies negatively, especially in relation to widely promoted societal ideals of beauty, it can lead to disordered eating habits and harmful behaviors [15]. These negative BI perceptions are linked to an increased risk of mental health issues, including depression, anxiety, and low self-esteem [16]. Moreover, body dissatisfaction is associated with higher levels of stress, which can impair sleep quality and affect social relationships, creating a cycle that exacerbates psychological health issues [17].

Physical activity (PA) has been widely recognized for its positive impact on mental health, particularly for adolescents, who are more vulnerable to emotional challenges [18,19,20]. By fostering self-esteem, reducing stress, and enhancing mood, PA offers a crucial pathway to improving mental well-being [18,19,20]. Studies, including one involving 1049 young adult men aged 18–29, demonstrate that diverse forms of PA correlate with better mental health outcomes [18]. However, challenges such as limited sample diversity and difficulties in establishing causality highlight the need for further investigation [18].

PA is particularly impactful in mediating the relationship between BI and mood disorders [21,22,23]. The Exercise and Self-Esteem Model (EXSEM) [24] and the Affect and Health Behavior Framework (AHBF) [25] provide valuable insights into the mechanisms by which PA can enhance mental health, particularly in adolescents. According to the EXSEM, engaging in PA improves self-esteem and self-worth, which positively influence body satisfaction and emotional regulation. This model highlights PA as a powerful tool for enhancing self-perception, especially for adolescents with negative BI perceptions [24,26]. The enhancement of self-esteem serves as a buffer against the onset of mood disorders, promoting emotional resilience [24,27]. Similarly, the AHBF underscores the importance of PA in regulating affective states through neurochemical changes such as endorphin release, which contributes to improved mood [25,27,28]. This framework also emphasizes the significance of positive emotional experiences during PA, suggesting that the psychological benefits of PA—such as feelings of accomplishment and social connection—are crucial for reducing stress and improving overall well-being [25,29].

Both frameworks underline the multifaceted nature of PA, highlighting its capacity to help adolescents develop coping mechanisms, regulate emotions, and navigate the challenges of adolescence more effectively [21,25,30]. PA is not only a preventive measure against the development of mood disorders but is also an intervention to enhance mental health outcomes [13,16,26,28]. By fostering a positive feedback loop, improvements in mood and self-esteem encourage more frequent participation in PA, further enhancing psychological well-being [16,23,31]. However, despite the theoretical strength of these models, reliance on self-reported data and the lack of longitudinal studies remain significant limitations in current research [20,21,23]. Self-reported measures, while useful, are subject to biases such as social desirability and recall errors [32]. Moreover, the absence of longitudinal studies hinders our ability to establish causal relationships and fully understand the long-term impact of PA on BI and mood disorders [20]. Future research should focus on overcoming these limitations to validate these theoretical frameworks and provide deeper insights into the mechanisms by which PA can benefit adolescent mental health [20,21].

Evidence further underscores the significance of the context in which PA is performed [19,20,28]. A study of 726 adolescents found that mental health benefits are maximized in supportive, enjoyable environments, emphasizing the social dimension of PA [19]. Additionally, a systematic review confirmed the physical, mental, and social advantages of PA for youth, while urging more inclusive and comprehensive research to fully capture its potential [20]. Also, that review study reinforced PA’s role as an essential intervention to enhance adolescent mental health and address the challenges associated with BI and mood disorders [20].

Despite these insights, there is a clear need for larger, representative studies to explore the nuanced relationships among PA, BI, and low mood in adolescents. This study had two main goals: (i) to examine the relationship between BI and low mood among adolescents, and (ii) to explore the role of PA as a mediator in this relationship. We hypothesized that PA mediates the relationship between BI dissatisfaction and low mood in adolescents. It is also hypothesized that there is a significant negative relationship between body image dissatisfaction and low mood in adolescents.

## 2. Methods

### 2.1. Study Design and Population 

This cross-sectional study used data from the Health Behaviour in School-aged Children (HBSC) survey [33], a collaborative effort with the World Health Organization. The HBSC is an international study aimed at monitoring and assessing the health and well-being of children and adolescents using a standardized research approach [34].

For this study, data from the 2017/2018 HBSC wave were analyzed. The dataset included nationally representative samples of school-aged children and adolescents, aged 10 to 17, from 43 countries. These countries included participants from Albania (*n* = 1291), Armenia (*n* = 2821), Austria (*n* = 3511), Azerbaijan (*n* = 3900), Belgium (Flemish) (*n* = 3473), Belgium (French) (*n* = 2945), Bulgaria (*n* = 3978), Canada (*n* = 6297), Croatia (*n* = 4211), the Czech Republic (*n* = 9581), Denmark (*n* = 2436), England (*n* = 281), Estonia (*n* = 3820), France (*n* = 6864), Georgia (*n* = 2406), Germany (*n* = 3550), Greece (*n* = 3308), Greenland (*n* = 386), Hungary (*n* = 3214), Iceland (*n* = 5231), Israel (*n* = 2440), Ireland (*n* = 749), Italy (*n* = 3378), Kazakhstan (*n* = 3216), Latvia (*n* = 4022), Lithuania (*n* = 3092), Luxembourg (*n* = 2785), Malta (*n* = 1340), the Netherlands (*n* = 3240), Poland (*n* = 4478), Portugal (*n* = 5409), the Republic of Moldova (*n* = 3946), Romania (*n* = 2651), the Russian Federation (*n* = 3600), Scotland (*n* = 1236), Serbia (*n* = 3245), Slovenia (*n* = 5228), Spain (*n* = 3790), Sweden (*n* = 2915), Switzerland (*n* = 6519), Ukraine (*n* = 5121), and Wales (*n* = 3741). Finland, North Macedonia, Norway, and Slovakia were excluded because data on body image or feelings of low mood were unavailable. A total of 154,183 participants (mean age = 13.6 years; standard deviation (SD) = 1.6; 51.5% girls) were included in the analysis. Participants were randomly selected from various schools and completed a standardized questionnaire anonymously in classroom settings, using their native languages. Students had the option to skip any questions they chose not to answer. Institutional ethical approval was obtained from each participating country, and written informed consent was provided by the schools, children, adolescents, and their parents or legal guardians. Since this study involved a secondary analysis of anonymized data, formal ethics committee approval was not required.

#### Measurements

Feeling low mood. Understanding that psychosomatic symptoms are key signs of mental health in adolescence, the international HBSC study developed the Symptom Checklist (SCL) [29,30]. This non-clinical tool checks how often young people experience eight common symptoms, including feelings of low mood. Feeling low was evaluated using the question “How often have you had low feelings?” Participants could choose from five response options: 1—about every day; 2—more than once/week; 3—about every week; 4—about every month; 5—rarely or never. The question related to feeling low or depressed demonstrated the highest regression coefficient (β = 0.80, *p* < 0.001) in relation to the HBSC-SCL psychological complaints factor [35]. This measure has exhibited acceptable reliability and internal consistency among adolescents of the same age group in Canada [35].

Physical activity. To assess PA, participants were asked, “In a typical week, on how many days do you do at least 60 min of physical activity?” with the response options ranging from 0 to 7 days per week [33]. The WHO recommends that children and adolescents engage in an average of 60 min of moderate to vigorous physical activity (MVPA) per day [36].

Body image. The BI item has been included in the HBSC study since the 1993/94 survey, where it was specifically developed for this purpose. Similar questions are also featured in other validated health-related questionnaires [31]. The BI item measures body dissatisfaction (BD) in relation to self-perceived body weight, which is important, because subjective well-being and weight-related behaviors are strongly associated with this aspect of BI [33,34]. The BI was assessed through the question: “Do you think your body is…?” Participants could select from five response options: “much too thin”, “a bit too thin”, “about the right size”, “a bit too fat”, and “much too fat”.

The adolescents provided self-reported data on their age and sex. They also reported their weight and height, which were used to calculate their body mass index (BMI) in kg/m^2^. BMI z-scores were then calculated according to the criteria set by the International Obesity Task Force [32]. This approach allows for determining the prevalence of thinness, normal weight, overweight, or obesity [37].

### 2.2. Statistical Analysis

In this study, categorical variables are presented as counts and percentages, while continuous variables are reported as means and standard deviations (SD), using listwise deletion methods to handle missing data. Multinomial logistic regression was used to determine the relationship of BI, PA, BMI, and sociodemographic factors with feelings of low mood.

In addition, we aimed to investigate the mediating role of PA in the relationship between BI and low mood. Linear regression models were fitted to estimate the effects: (a) the effect of BI on PA (*BI* -> *PA*), (b) the direct effect of BI on low mood (*BI* -> low mood), and (c) the effect of PA on low mood (*PA* -> low mood). The mediation analysis was performed using the mediation package in R. Specifically, the average causal mediation effect (ACME), the average direct effect (ADE), the total effect, and the proportion mediated were estimated with confidence intervals based on 1000 bootstrap simulations.

The adjusted regression coefficients were used to construct a path diagram representing the relationships among the variables. The diagram was generated with the assistance of the semPlot package (version 1.1.6). The analysis was conducted using RStudio (version 2023.12.1+402), Boston, Massachusetts, USA. The arrows in the diagram indicate the direct and mediating paths, accompanied by the estimated values for ACME, ADE, and the total effect, including their confidence intervals and significance levels [38].

To reduce potential bias from missing data, we applied multiple imputation techniques as part of a sensitivity analysis. Missing values, which refer to data points that are unknown, uncollected, or incorrectly entered, were handled by removing any extraneous whitespace from the dataset. Fields and records containing missing values were excluded as needed [39].

## 3. Results

Adolescents who regularly practiced PA (7 days a week) had a lower prevalence of low mood: 28.5% for the 10–12 age group and 33.6% for the 13–15 age group. Boys accounted for 48.5% of participants and tended to do more PA (59.1%) than girls (40.9%). The majority of adolescents (70.3%) were in the normal weight category, which linked with lower mood, while those who are overweight (12.8%) and obese (2.8%) had higher rates. Positive BI was associated with lower rates of low mood, especially among those who perceived themselves to be of ideal weight (56.1%) (Table 1).

**Figure 1:**
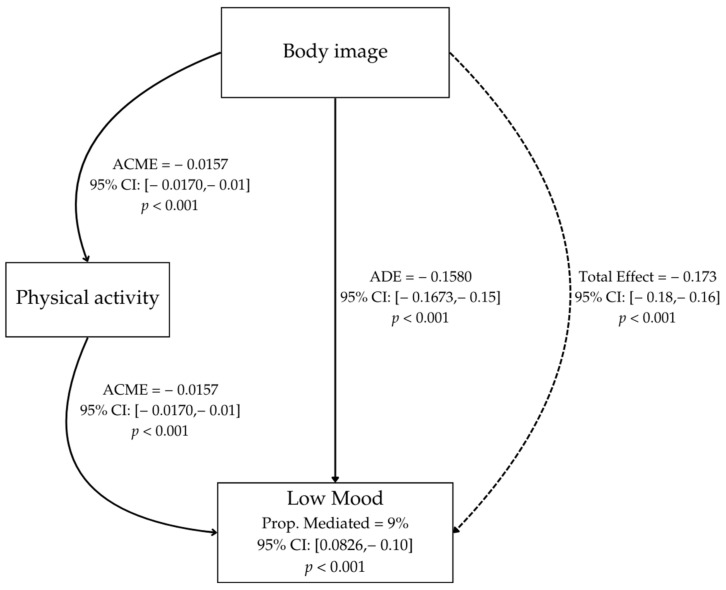
Mediation analysis among feeling low mood, body image, and physical activity. ACME = average causal mediation effect, ADE = average direct effect. **Solid arrows** represent direct pathways where one variable influences another. **Dashed arrows** represent indirect or total effects that take into account mediation effects.

The results revealed a significant mediated effect of PA in the relationship between BI and low mood (*p* < 0.001), although its contribution is relatively small (see Figure 1). Additionally, the direct effect of BI on LM was strong and negative (*p* < 0.001), as was the total effect (*p* < 0.001), showing that a negative BI directly and significantly impacts low mood. This total effect encompasses both direct and mediated pathways. The proportion of the total effect mediated by PA was approximately 9% (*p* < 0.001). These findings suggest that PA plays a partial mediating role in the relationship between BI and LM, although most of the effect of BI on LM is direct (Figure 1). 

The multinomial logistic regression revealed that daily PA significantly reduces the risk of low mood, as seen in the “about every day” category [PA on 6 days (odds ratio (OR) = 0.72); PA on 5 days (OR = 0.86); PA on 4 days (OR = 0.91)]. On the other hand, in the “more than once a week” category, the risk decreases as PA frequency increases (PA on 5 days (OR = 1.17); PA on 4 days (OR = 1.29); PA on 3 days (OR = 1.50)). Finally, in the “about every week” and “about every month” low mood categories, individuals engaging in PA less than three days per week still show an increased risk of low mood (PA on 2 days (OR = 1.59); PA on 1 day (OR = 1.68); PA on 0 days (OR = 1.44)) (Table 2).

Age, BMI, BI perception, and sex are associated with the frequency of low mood. Individuals who perceive themselves as “much too thin”, “a bit too thin”, “about right”, or “a bit too fat” have lower odds of experiencing low mood compared with those who perceive themselves as “much too fat”, with ORs ranging from 0.12 to 0.50 (Table 3). Age is positively associated with low mood feelings, with the OR being highest for those reporting low mood “almost every day” (OR = 1.21) and “more than once a week” (OR = 1.20) (Table 4). Males are less likely to report low mood than females, with ORs ranging from 0.40 (“almost every day”) to 0.77 (“about once a month”) (Table 4). Regarding BMI, individuals classified as “thinness”, “normal weight”, or “overweight” show an increased risk of low mood feelings compared with those with “obesity”, with the strongest association observed in the “thinness” category (e.g., OR = 1.32, *p* = 0.001 for “almost every day”). The relationship between overweight and low mood is weaker and not statistically significant in some categories (e.g., OR = 1.09, *p* = 0.114 for “more than once a week”) (Table 4).

## 4. Discussion

Our study found that PA partially mediates the relationship between BI and low mood, although its contribution remains relatively small. While PA helps mitigate the negative impact of BI on mood, the majority of the relationship appears to be direct. These findings suggest that interventions aimed at improving BI and promoting PA could help reduce the risk of low mood in adolescents. Previous research supports this, showing that PA serves as a positive outlet for stress, enhances body satisfaction, and helps adolescents develop resilience to social pressures [9,10,22,40]. The EXSEM [24] and the AHBF [25] provide valuable insights into these mechanisms. The EXSEM explains how PA contributes to improved self-esteem through physical competence and body image satisfaction, which, in turn, positively impacts mood [23,24,26]. The AHBF highlights the role of affective responses in shaping health behaviors, suggesting that regular PA promotes positive emotional states, reinforcing engagement in health-protective behaviors [23,25].

An inverse relationship between PA and low mood was evident, demonstrating that the frequency of PA plays a crucial role in mental well-being. Regular exercise—especially five or more days per week—had a significant protective effect, reducing the risk of low mood, as seen in the “about every day” category. Conversely, irregular or insufficient exercise failed to provide the same protective effect and, in some cases, was associated with an increased risk of low mood. These findings align with Samsudin et al. [41], who emphasized PA’s role in reducing depressive symptoms in adolescents. Similarly, Pearce et al. [42] found a strong association between PA and a lower risk of depression, reinforcing the importance of regular exercise as a preventive and therapeutic strategy for mental health. These studies highlight that even small variations in PA frequency can significantly impact feelings of low mood [5,13,23].

Consistent with prior studies, PA is associated with improved self-esteem, reduced stress, and better overall mood [13,18,19,22,43]. It is well-documented that regular PA can alleviate depressive symptoms and enhance body satisfaction among adolescents, acting as a buffer against negative BI and low mood [19,20,22,43]. Our study overcomes the limitations of previous research, such as small sample sizes and limited geographical scope, by using a large, diverse sample of over 150,000 participants, which offers a more comprehensive understanding of the relationships among PA, BI, and low mood [31].

Additionally, our findings suggest BI perception has a stronger influence on low mood than BMI alone. Adolescents who perceived themselves as “much too fat” were at the highest risk of experiencing low mood. For instance, in the “almost every day” category, those who perceived themselves as “about right” had a significantly lower likelihood of reporting low mood feelings (OR = 0.12). Interestingly, the association was strongest for those classified as underweight, with a higher OR, indicating that having a low BMI may be linked to an increased probability of experiencing low mood. In contrast, the relationship with overweight was weaker and, in some categories, not statistically significant (e.g., OR = 1.09, *p* = 0.114 for “more than once a week”). Additionally, adolescents with a normal BMI exhibited the lowest rates of low mood, while those with overweight or obesity, even when physically active, still reported elevated levels of low mood symptoms [4,8,13,15]. These findings underscore the importance of addressing BI perception in PA programs, as improving body image satisfaction may be crucial for enhancing mental health outcomes, particularly among adolescents with higher BMI [13,15].

Our study found a positive association between age and the frequency of low mood, with older adolescents reporting higher rates of low mood feelings. This may reflect increasing psychosocial stressors and BI concerns as adolescents mature. Given this trend, PA could serve as a valuable tool for improving self-esteem and mitigating mood-related issues as adolescents age [15,17].

Sex differences were also evident, with males consistently reporting lower odds of experiencing low mood compared with females. This finding aligns with previous research suggesting that girls face greater societal and cultural pressures related to BI, which may contribute to their heightened vulnerability to mood disturbances [2,4,11,44]. These results highlight the need for sex-specific interventions that address the unique challenges faced by adolescent girls, particularly in relation to body satisfaction and mental well-being.

These insights emphasize the importance of integrating PA into public health strategies to enhance adolescent mental health, particularly considering the associations with age, sex, and BI perception. School-based PA programs that promote body positivity and self-acceptance could be more effective in engaging adolescents struggling with BI concerns [2,13]. Furthermore, fostering a supportive community and family environment is essential to reinforcing the positive impact of PA on adolescent mood and self-perception [5,31].

Future research should adopt longitudinal designs to better understand the causal relationships among PA, BI, and low mood over time [10]. Investigating cultural and societal influences on BI perception could provide additional insights, as cultural norms heavily shape adolescents’ views of themselves [5]. Further exploration of the long-term effects of PA on mental health as adolescents transition into adulthood may offer valuable information on the sustained benefits of early, positive PA habits for lifelong well-being [13].

### Limitations and Strengths

While this study is cross-sectional and thus cannot establish causality, it leverages a large and diverse sample, enhancing the generalizability of the results. Self-reported data may introduce bias, particularly regarding BI, PA, and low mood, but our use of a well-established international dataset (HBSC) mitigates this to some extent by standardizing measurements across a broad population.

## 5. Conclusions

PA partially mediates the relationship between BI and low mood, with a direct negative impact of BI on low mood. In addition, the study highlights that girls, older adolescents, and those with negative BI and irregular PA have a higher risk of low mood. Interventions that improve BI can directly reduce levels of low mood in adolescents, and part of this benefit occurs through improvements in PA.

## Figures and Tables

**Table 1:** Descriptive data of the study participants according to frequency of physical activity (days) by the listwise deletion method (*n* = 154,183).

Variables		Frequency of Physical Activity (Days)								
		0	1	2	3	4	5	6	7	Total (%)
Age group (%)	Aged 10–12 years	1570 (21.2)	2882 (26.2)	5149 (26.7)	7352 (28.5)	7396 (30.6)	7530 (32.3)	4991 (33.1)	10,665 (37.8)	47,535 (30.8)
	Aged 13–15 years	2220 (29.9)	3471 (31.5)	6579 (34.2)	8886 (34.5)	8616 (35.6)	8131 (34.9)	5338 (35.4)	9479 (33.6)	52,720 (34.2)
	Aged 16–17 years	3626 (48.9)	4656 (42.3)	7529 (39.1)	9527 (35.0)	8160 (33.8)	7655 (32.8)	4741 (31.5)	8034 (28.5)	53,928 (35.0)
Sex (%)	Boy	3093 (41.7)	4335 (39.4)	7598 (39.5)	11,324 (44.0)	11,623 (48.1)	12,084 (51.8)	8048 (53.4)	16,663 (59.1)	74,768 (48.5)
	Girls	4323 (58.3)	6674 (60.6)	11,659 (60.5)	14,441 (54.0)	12,549 (51.9)	11,232 (48.2)	7022 (46.6)	11,515 (40.9)	79,415 (51.5)
BMI (%)	Thinness	1122 (15.1)	1639 (14.9)	2275 (14.4)	3538 (13.7)	3281 (13.6)	3134 (13.4)	2056 (13.6)	4367 (15.5)	21,912 (14.2)
	Normal weight	4820 (65.0)	7357 (66.8)	13,016 (67.6)	17,730 (68.8)	17,084 (70,7)	16,789 (72.0)	11,181 (74.2)	20,353 (72.2)	108,330 (70.3)
	Overweight	1159 (15.6)	1611 (14.6)	2754 (14.3)	3685 (14.3)	3114 (12.9)	2866 (12.3)	1574 (10.4)	2909 (10.3)	19,672 (12.8)
	Obesity	315 (4.2)	402 (3.7)	712 (3.7)	812 (3.2)	693 (2.9)	527 (2.3)	259 (1.7)	549 (1.9)	4269 (2.8)
Feeling low mood (%)	About every day	1278 (17.2)	1340 (12.2)	2016 (10.5)	2078 (8.1)	1662 (6.9)	1507 (6.5)	816 (5.4)	2074 (7.4)	12,771 (8.3)
	More than once/week	1090 (14.7)	1729 (15.7)	2475 (12.9)	2123 (12.2)	2529 (10.5)	2234 (9.6)	1312 (8.7)	2298 (8.2)	16,799 (10,9)
	About every week	949 (12.8)	1565 (14.2)	2752 (14.3)	3549 (13.8)	3223 (13.3)	2799 (12.0)	1839 (12.2)	2805 (10.0)	19,481 (12.6)
	About every month	1205 (16.2)	2092 (19.0)	4084 (21.2)	5579 (21.7)	5401 (22.3)	5241 (22.5)	3337 (22.1)	5229 (18.6)	32,168 (20.9)
	Rarely or never	2894 (39.0)	4283 (38.9)	7930 (41.2)	11,427 (44.4)	11,357 (47.0)	11,535 (49.5)	7766 (51.5)	15,772 (56.0)	72,964 (47.3)
Body image (%)	Much too thin	401 (5.4)	453 (4.1)	716 (3.7)	718 (2.8)	705 (2.9)	665 (2.9)	423 (2.8)	1290 (4.6)	5371 (3.5)
	Bit too thin	1125 (15.2)	1521 (13.8)	2725 (14.2)	3625(14.1)	3391 (14.0)	3304 (14.2)	2286 (15.0)	4338 (15.4)	22,315 (14.5)
	About right	3454 (46.6)	5671 (51.5)	9903 (51.4)	13,833 (55.7)	13,467 (55.7)	13,543 (58.1)	9092 (60.3)	17,603(62.5)	86,569 (56.1)
	Bit too fat	1903 (25.7)	2856 (25.9)	5126 (26.6)	6614 (25.7)	5882 (24.3)	5155 (22.1)	2920 (19.4)	4301 (15.3)	34,757 (22.5)
	Much too fat	530 (7.1)	508 (4.6)	787 (4.1)	975 (3.8)	727 (3.0)	649 (2.8)	349 (2.3)	646 (2.3)	5171 (3.4)

**Table 2:** Multinomial logistic regression analysis for predictors of feeling low mood on physical activity.

Low Mood Category ^a^	Variable Predictor	Beta	OR (95% IC)	*p*-Value
About every day	PA 0 days	0.78	2.19 (2.01–2.38)	<0.001
	PA 1 days	0.52	1.69 (1.56–1.83)	<0.001
	PA 2 days	0.32	1.38 (1.29–1.48)	<0.001
	PA 3 days	0.05	1.05 (0.98–1.12)	0.13
	PA 4 days	−0.09	0.91(0.84–0.97)	0.01
	PA 5 days	−0.15	0.86 (0.80–0.92)	<0.001
	PA 6 days	−0.32	0.72 (0.66–0.79)	<0.001
	PA 7 days	0	0	0
More than once/week	PA 0 days	0.61	1.84 (1.69–2.01)	<0.001
	PA 1 days	0.74	2.10 (1.95–2.25)	<0.001
	PA 2 days	0.48	1.62 (1.52–1.73)	<0.001
	PA 3 days	0.40	1.50 (1.41–1.59)	<0.001
	PA 4 days	0.25	1.29 (1.21–1.37)	<0.001
	PA 5 days	0.16	1.17 (1.10–1.25)	<0.001
	PA 6 days	0.05	1.05 (0.98–1.14)	0.12
	PA 7 days	0	0	0
About every week	PA 0 days	0.36	1.44 (1.32–1.57)	<0.001
	PA 1 days	0.52	1.68 (1.56–1.80)	<0.001
	PA 2 days	0.46	1.59 (1.50–1.69)	<0.001
	PA 3 days	0.39	1.47 (1.39–1.56)	<0.001
	PA 4 days	0.34	1.40 (1.32–1.48)	<0.001
	PA 5 days	0.21	1.24 (1.17–1.31)	<0.001
	PA 6 days	0.21	1.24 (1.16–1.32)	<0.001
	PA 7 days	0	0	0
About every month	PA 0 days	0.09	1.10 (1.02–1.18)	0.010
	PA 1 days	0.28	1.32 (1.24–1.40)	<0.001
	PA 2 days	0.33	1.39 (1.32–1.46)	<0.001
	PA 3 days	0.29	1.34 (1.28–1.40)	<0.001
	PA 4 days	0.29	1.33 (1.27–1.40)	<0.001
	PA 5 days	0.26	1.30 (1.24–1.36)	<0.001
	PA 6 days	0.21	1.24 (1.18–1.31)	<0.001
	PA 7 days	0	0	0

^a^ The reference category is rarely or never. PA, physical activity.

**Table 3:** Multinomial logistic regression analysis for predictors of feeling low mood on body image.

Low Mood Category ^a^	Variable Predictor	Beta	OR (95% IC)	*p*-Value
About every day	BI—Much too thin	−1.09	0.33 (0.29–0.37)	<0.001
	BI—Bit too thin	−1.75	0.17 (0.15–0.19)	<0.001
	BI—About right	−2.11	0.12 (0.11–0.13)	<0.001
	BI—Bit too fat	−1.26	0.28 (0.25–0.30)	<0.001
	BI—Much too fat	0	0	0
More than once/week	BI—Much too thin	−0.81	0.44 (0.32–0.50)	<0.001
	BI—Bit too thin	−1.07	0.34 (0.30–0.37)	<0.001
	BI—About right	−1.34	0.26 (0.23–0.28)	<0.001
	BI—Bit fat	−0.67	0.50 (0.46–0.55)	<0.001
	BI—Much too fat	0	0	0
About every week	BI—Much too thin	−0.58	0.55 (0.48–0.63)	<0.001
	BI—Bit too thin	−0.57	0.56 (0.50–0.62)	<0.001
	BI—About right	−0.85	0.42 (0.38–0.46)	<0.001
	BI—Bit too fat	−0.33	0.71 (0.64–0.79)	<0.001
	BI—Much too fat	0	0	0
About every month	BI—Much too thin	−0.45	0.63 (0.56–0.71)	<0.001
	BI—Bit too thin	−0.26	0.76 (0.69–0.84)	<0.001
	BI—About right	−0.40	0.66 (0.60–0.73)	<0.001
	BI—Bit too fat	−0.10	0.90 (0.82–0.99)	0.03
	BI—Much too fat	0	0	0

^a^ The reference category is rarely or never. BI, body image.

**Table 4:** Multinomial logistic regression analysis for predictors of feeling low mood on demographic variables (age, sex, BMI).

Low Mood Category ^a^	Variable Predictor	Beta	OR (95% IC)	*p*-Value
About every day	Age	0.19	1.21 (1.20–1.23)	<0.001
	Male	−0.90	0.40 (0.38–0.42)	<0.001
	Female	0	0	0
	BMI—thinness	0.27	1.32 (1.16–1.49)	<0.001
	BMI—normal weight	0.25	1.29 (1.15–1.44)	<0.001
	BMI—overweight	0.17	1.19 (1.06–1.34)	0.003
	BMI—obesity	0	0	0
More than once/week	Age	0.18	1.20 (1.18–1.21)	<0.001
	Male	−0.75	0.46 (0.45–0.48)	<0.001
	Female	0	0	0
	BMI—thinness	0.18	1.19 (1.06–1.34)	0.002
	BMI—normal weight	0.18	1.20 (1.08–1.33)	<0.001
	BMI—overweight	0.08	1.09 (0.97 -1.21)	0.114
	BMI—obesity	0	0	0
About every week	Age	0.15	1.16 (1.15–1.17)	<0.001
	Male	−0.53	0.58 (0.56–0.60)	<0.001
	Female	0	0	0
	BMI—thinness	0.17	1.18 (1.05–1.32)	0.003
	BMI—normal weight	0.23	1.26 (1.14 -1.40)	<0.001
	BMI—overweight	0.13	1.14 (1.02 -1.27)	0.016
	BMI—obesity	0	0	0
About every month	Age	0.09	1.10 (1.09–1.11)	<0.001
	Male	−0.25	0.77 (0.75–0.79)	<0.001
	Female	0	0	0
	BMI—thinness	0.22	1.24 (1.13–1.37)	<0.001
	BMI—normal weight	0.20	1.23 (1.12–1.34)	<0.001
	BMI—overweight	0.12	1.13 (1.03–1.24)	<0.001
	BMI—obesity	0	0	0

^a^ The reference category is rarely or never. BMI, body mass index.

## Data Availability

The HBSC database is publicly available. Click here to access it: https://hbsc.org/data/.
